# Feasibility of the automatic ingestion monitor (AIM-2) for infant feeding assessment: a pilot study among breast-feeding mothers from Ghana

**DOI:** 10.1017/S1368980022001264

**Published:** 2022-10

**Authors:** Caroline Cerminaro, Edward Sazonov, Megan A McCrory, Matilda Steiner-Asiedu, Viprav Bhaskar, Sina Gallo, Emma Laing, Wenyan Jia, Mingui Sun, Tom Baranowski, Gary Frost, Benny Lo, Alex Kojo Anderson

**Affiliations:** 1 Department of Nutritional Sciences, University of Georgia, 280 Dawson Hall, 305 Sanford Drive, Athens, GA 30602, USA; 2 Department of Electrical and Computer Engineering, University of Alabama, Tuscaloosa, AL, USA; 3 Department of Health Sciences, Boston University, Boston, MA, USA; 4 Department of Nutrition and Food Science, University of Ghana, Legon-Accra, Ghana; 5 Department of Electrical and Computer Engineering, University of Pittsburgh, Pittsburgh, PA, USA; 6 Department of Neurological Surgery, University of Pittsburgh, Pittsburgh, PA, USA; 7 USDA/ARS Children’s Nutrition Research Center, Baylor College of Medicine, Houston, TX, USA; 8 Department of Medicine, Imperial College London, London, UK; 9 The Hamlyn Center, Imperial College London, London, UK

**Keywords:** Automatic Ingestion Monitor, Breast-feeding, Infant feeding assessment, Wearable technology

## Abstract

**Objective:**

Passive, wearable sensors can be used to obtain objective information in infant feeding, but their use has not been tested. Our objective was to compare assessment of infant feeding (frequency, duration and cues) by self-report and that of the Automatic Ingestion Monitor-2 (AIM-2).

**Design:**

A cross-sectional pilot study was conducted in Ghana. Mothers wore the AIM-2 on eyeglasses for 1 d during waking hours to assess infant feeding using images automatically captured by the device every 15 s. Feasibility was assessed using compliance with wearing the device. Infant feeding practices collected by the AIM-2 images were annotated by a trained evaluator and compared with maternal self-report via interviewer-administered questionnaire.

**Setting:**

Rural and urban communities in Ghana.

**Participants:**

Participants were thirty eight (eighteen rural and twenty urban) breast-feeding mothers of infants (child age ≤7 months).

**Results:**

Twenty-five mothers reported exclusive breast-feeding, which was common among those < 30 years of age (*n* 15, 60 %) and those residing in urban communities (*n* 14, 70 %). Compliance with wearing the AIM-2 was high (83 % of wake-time), suggesting low user burden. Maternal report differed from the AIM-2 data, such that mothers reported higher mean breast-feeding frequency (eleven *v*. eight times, *P* = 0·041) and duration (18·5 *v*. 10 min, *P* = 0·007) during waking hours.

**Conclusion:**

The AIM-2 was a feasible tool for the assessment of infant feeding among mothers in Ghana as a passive, objective method and identified overestimation of self-reported breast-feeding frequency and duration. Future studies using the AIM-2 are warranted to determine validity on a larger scale.

Increases in breast-feeding incidence and duration can improve health of the global population. Breast-feeding allows mothers to physically connect with their infant and adapt to his or her needs. Aside from the engagement that occurs between mother–infant dyads, breast-feeding provides a plethora of health, economic and developmental benefits to mother and baby^(1–3)^. Health benefits for the mother are both physical and emotional, ranging from decreased diabetes risk to oxytocin release that causes the uterus to shrink to its pre-pregnancy state after childbirth^(4–6)^. Infant benefits are similarly extensive, ranging from brain development to protection against gastrointestinal disorders^(7–9)^. The WHO and UNICEF recommend to exclusively breast-feed for 6 months without any food or water supplementation. Exclusive breast-feeding is particularly crucial in low- and middle-income countries with higher prevalence of poverty and undernutrition due to its economic and health benefits^(10)^. Despite progress in Africa, the percentage of infants meeting the recommendation for exclusive breast-feeding for 6 months remains under 20 % in some African countries^(11)^.

Multiple barriers have been reported that prevent mothers from meeting breast-feeding recommendations^(12)^. Perceived insufficient milk supply is one of the common reasons for discontinuation of breast-feeding in both nourished and undernourished populations^(13)^. Infant satisfaction cues are often misinterpreted by mothers as the chief indication of milk supply^(13)^. Yet, primary glandular insufficiency, the main reason for insufficient milk supply, occurs in <5 % of all women^(14)^. Most early breast-feeding problems can be prevented with early latching within the first hour of life and feeding often on the earliest signs of hunger, or quickly resolved with appropriate support, including education about normal patterns and volumes of newborn feeds.

Previously published studies have attempted to unveil the difficulties of infant feeding and reasons for early cessation^(15–17)^. Most of the existing studies have relied on maternal report with no active observation or objective assessment^(10,12)^. Memory-based recall, especially regarding dietary habits, has been widely questioned as an accurate means of data collection^(18–20)^. Women, including mothers of young infants, have generally been found to underreport their own energy intake and overreport intake of their children^(18,21)^. This bias poses challenges to researchers attempting to study infant feeding habits and how they influence developmental and health outcomes as well as practitioners during patient support. Test weighing is considered a gold standard method of assessing infant breast milk intake, including frequency, duration and amount, but is burdensome, especially if frequency and duration are of primary interest.

One proposed alternative for more accurate assessment of infant feeding habits does not rely on maternal memory or recall and imparts relatively low burden on the mother. This promising option is a wearable device for image-based dietary assessment, which is both objective, not requiring self-report, and passive, not requiring participant involvement^(22–24)^. Images captured by a passive device, i.e. not requiring active input from the user, overcome the issue of participant bias and provide researchers a frame-by-frame view of parent and child activities during dietary intake sessions. This technology-based method has been explored in dietary assessment studies, but never for the purpose of infant feeding assessment in low- and middle-income countries^(24–26)^. Further, geographic differences in feeding practices between urban and rural communities have not been examined using such an innovative method. Therefore, the purpose of the current study was to evaluate the feasibility of a wearable, image-capturing device, the Automatic Ingestion Monitor-2 (AIM-2), for infant feeding assessment among breast-feeding mothers in both rural and urban Ghanaian communities by (a) assessing the user compliance with the AIM-2 device for this purpose and (b) comparing caregiver-reported and AIM-2 captured infant feeding patterns.

## Methods

This cross-sectional study was conducted in Accra in the Greater Accra Region and Mampong-Akuapem in the Eastern Region of Ghana, Africa, among mothers of young infants using a mixed-methods approach. Data collection took place from October 2019 to January 2020. Participants were recruited by word-of-mouth by trained field research assistants during their visit to rural and urban communities. Participants were recruited from their homes via house-to-house visit by trained field research assistants in the respective communities. Exclusive breast-feeding *v*. mixed feeding rates were determined via maternal self-report and AIM-2 captured images. Maternal reported exclusive breast-feeding was defined as feeding the infant breastmilk without any additional food or fluids, including water^(27)^, and image-based exclusive breast-feeding was based on the infant observed in the captured AIM-2 images to only feeding at the breast during the day of data collection. Infants observed to be feeding from a bottle, cup, spoon in addition to breast-feeding in the captured AIM-2 images were classified as mixed feeding. Participant inclusion was solely based on whether mother had a breast-feeding infant younger than 7 months old. Mothers of twins and other higher order births were excluded. Data were collected using both the AIM-2 device and a face-to-face interviewer-administered questionnaire. The interviewer-administered questionnaire collected information on caregiver-reported infant feeding practices, breast-feeding experiences and general demographic information such as occupation, level of education and marital status, among other characteristics. The AIM-2 device mounted on eyeglasses and worn by mothers during wake-time on a chosen day (day of data collection) was used for image capture. Captured images were processed and uploaded to MatLab 2020b (Mathworks) and custom-made AIM annotation software for annotation. All methods and procedures were reviewed and approved by the Human Subject Institutional Review Boards of the University of Georgia and the Noguchi Memorial Institute for Medical Research of the University of Ghana. All participants provided informed consent before recruitment for study participation. Participants also gave written consent for their photographs to be used in publication. Monetary incentives ($20) for participation were provided equally for each mother.

### Development and administration of the infant feeding questionnaire

The questionnaire was developed based on existing instruments and commonly reported breast-feeding challenges reported in the literature^(24,28–30)^. The questionnaire contained both closed- and open-ended questions. The yes/no portion of the questionnaire contained thirteen commonly reported breast-feeding struggles, including an infant’s difficulty sucking or overall disinterest in feeding^(28–30)^. The open-ended questions highlighted some of the more complex breast-feeding struggles, inquiring about overall experiences and concerns with breast-feeding. The questionnaire was pilot tested with ten mothers of young infants in Accra, Ghana, and revised with feedback from the mothers. The final questionnaire included the following categories: socio-demographic factors (*n* 12), current breast-feeding practices (*n* 4), open-ended breast-feeding experiences and concerns (*n* 7), possible breast-feeding problems (*n* 14) and open-ended physical observations before, during and after feeding (*n* 5). Infant feeding at the time of the survey was assessed with the question ‘How are you currently feeding your infant? [ ] Exclusive Breast-feeding [ ] Mixed-feeding (breast milk and other foods.’ For frequency of breast-feeding, we asked an open-ended question ‘How frequently have you been breast-feeding your baby on average each day?’ For duration of breast-feeding session, the participant was asked the question ‘On average, how long does each breast-feeding session last?’

Trained field research assistants administered the questionnaire in a face-to-face interview format in the participants’ home the day of data collection. The open-ended questions aimed to ascertain mother’s breast-feeding self-efficacy as well as breast-feeding challenges and successes. The closed-ended, yes/no questions about breast-feeding problems served to highlight common breast-feeding issues in the mothers’ experience. Potential problems included cracked or sore nipples, clogged milk ducts and struggle with milk flow^(31)^.

### Image capture with automatic ingestion monitor

**Fig. 1:**
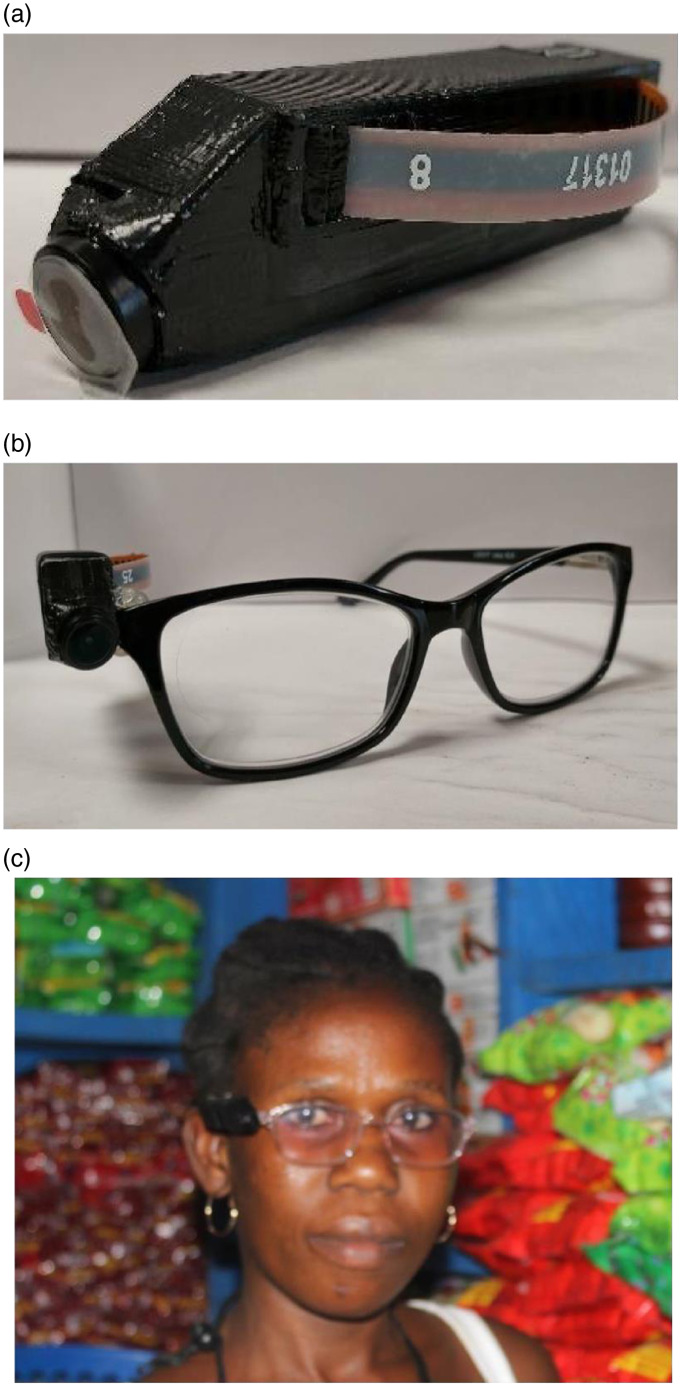
Automatic ingestion monitor

Before data collection with the AIM-2 (Fig. 1), a brief training session was conducted by the field research assistant with participants. The training session addressed how to properly wear the AIM-2, take the device off and on and how to charge the device if needed. The training session allowed participants to ask any initial questions about the device and its operation before data collection commenced. The AIM-2 mounted on eyeglasses was worn by mothers during wake time (from when mother woke up in the morning through when she was ready to go to bed) on the day of data collection. Participants were instructed to take-off the eyeglasses whenever they engaged in activities where they wanted to preserve privacy, such as using the bathroom, toileting and napping). Participants who normally wear prescription eyeglasses had the AIM-2 attached to their eyeglasses; all other participants were provided with non-corrective eyeglasses with AIM-2 attached. The AIM-2 automatically captured images every 15 s during wake time (Fig. 1).

The captured images were downloaded from the AIM-2 at the research lab, processed and uploaded to MatLab 2020b and a custom-made AIM annotation software. Images were manually annotated to determine type of feeding, breast-feeding duration, frequency and feeding cues of infant feeding sessions. All the image annotations were completed by CC after initial training. Sets of images from two random participants (one rural and one urban) were selected and coded separately by CC and AKA. The coding by CC was compared with that of AKA, discussed where there was disagreement and re-coded until we achieved agreement. We developed a Standard Operating Procedure for coding for the various infant cues used for coding. During the coding, whenever a new cue was encountered, it was discussed between CC and AKA, agreed on a code and added to the coding diary. User compliance was determined comparing ‘active’ to ‘inactive’ wear by participants during wake time. Compliant use of the AIM-2 was defined as when the participant wore the device as prescribed during wake time as measured by compliance detection software. Non-compliant was defined as when the device was not worn by the participant during wake-time or the device was worn incorrectly; for example, the device was placed on a table or over the forehead.

**Fig. 2:**
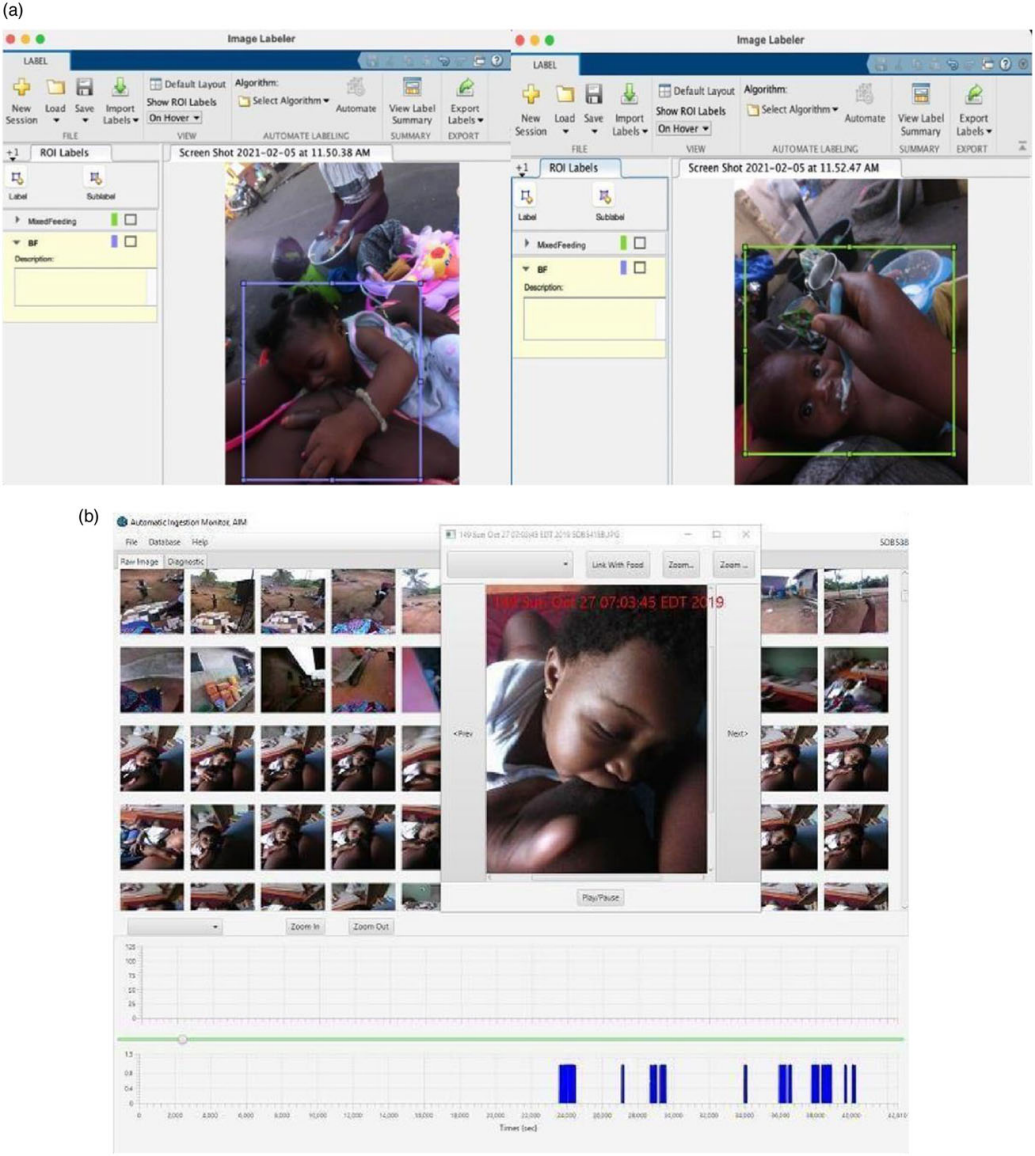
Infant feeding pattern analysis

In the MatLab 2020b software, human observer annotation was performed by a trained research assistant (CC) who was not part of the interview data collection using the Image Labeler Toolbox, after training by VB. This feature allowed viewing and manually identifying breast-feeding or mixed feeding sessions within each participant’s captured images during wear time. The cursor was used to outline the baby and method of feeding (either at the breast or food product) in colour-coded boxes (Fig. 2(a)). The AIM annotation software was used to assess breast-feeding duration using image time stamps (calculated as the time difference between the first and last images) per session, frequency and annotate feeding cues using infant facial expression (Fig. 2(b)). The beginning of feeding was defined when the infant latched on the breast, while the end of feeding was defined as when the infant came off the breast and did not latch back within 2 min as detected in the images or when the infant’s eyes was closed for at least 2 min without change in posture as observed from the images during a feeding session.

**Fig. 3:**
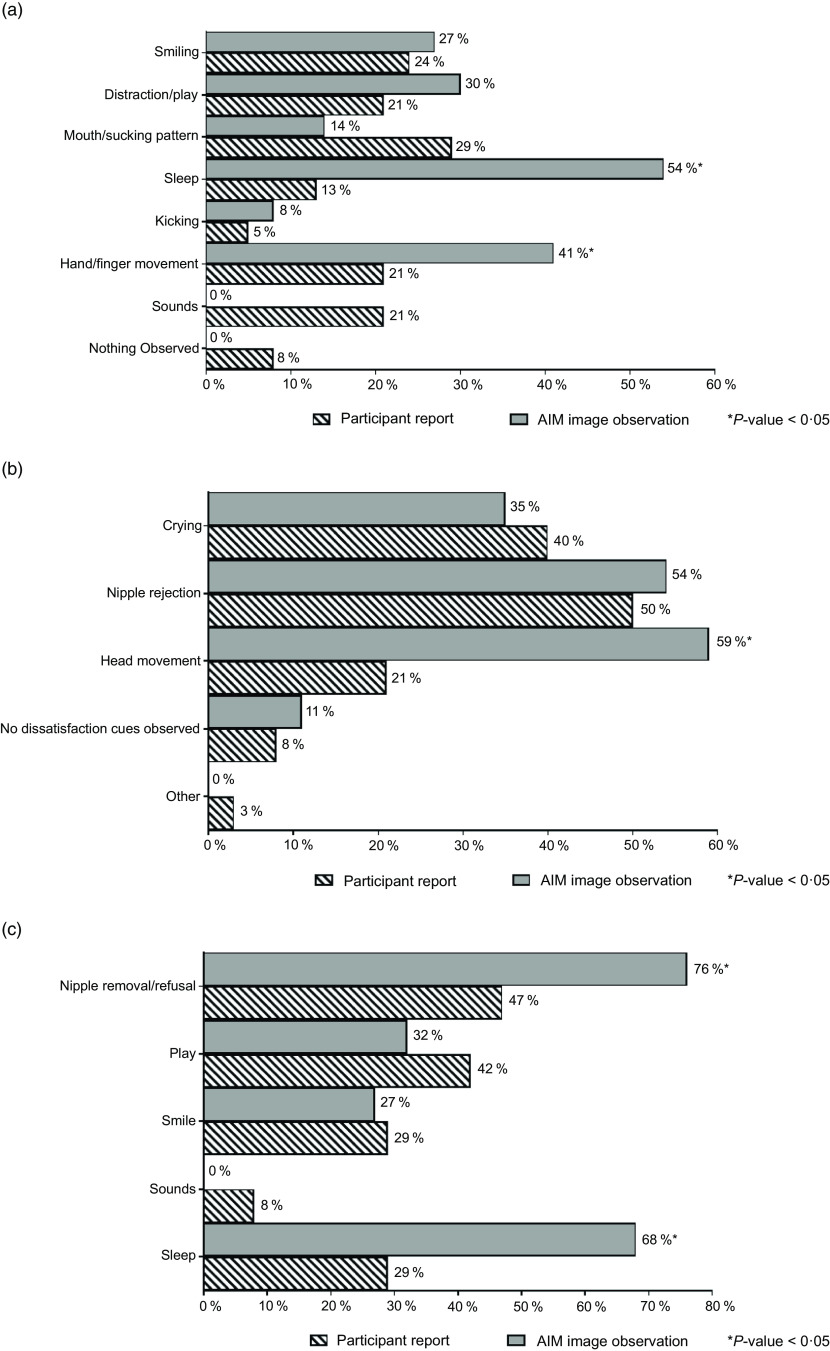
Maternal-reported *v*. researcher-observed infant feeding cues: during breast-feeding session

Feeding cues, which were either maternal-reported or observed in AIM images, were coded and categorised using key words. The cues were grouped based on whether they occurred before, during or at the end of the feeding and included the following categories: expression of hunger, expression of readiness to breast-feed, enjoyment/satisfaction cues, dissatisfaction/disinterest cues and expression of fullness/satiation, as agreed on by CC and AKA. Feeding cues occurring throughout the middle to end of feeding sessions are presented collectively as ‘during BF session’ (Fig. 3). Mothers reported diverse range of feeding cues, involving the infants’ head, hands, sounds and facial expressions, whereas CC coded similar cues from the captured images (as researcher observed).

### Data analysis

Descriptive analyses were performed using SPSS 27 (IBM) and reported as frequencies and percentages for categorical variables and means for continuous variables. Feasibility of the device as defined by participant compliance was assessed by the amount of time the device was actively worn *v*. taken off throughout the wake time. A comparison was performed between the captured images and questionnaire responses to evaluate the accuracy of mothers’ self-reported breast-feeding practices (exclusive breast-feeding *v*. mixed feeding, duration and frequency of breast-feeding). Accuracy of maternal report was defined as the percent of agreement or discrepancy with the AIM-2 image observations and determined for type of infant feeding, frequency, duration and feeding cues of breast-feeding. Feeding cues, which were either maternal-reported or observed in AIM images, were coded, and categorised using key words and compared. The cues were grouped based on whether they occurred before, during or at the end of the feeding session.

Maternal report of feeding frequency, duration and cues was compared between rural and urban participants and exclusive *v*. mixed feeding groups and determined by *χ*^2^ or Fisher’s exact test. Criterion for statistical significance was set as *P* < 0·05.

## Results

Thirty-eight mothers (eighteen rural and twenty urban) and their infants under 7 months of age participated in the study. Participant age ranged from 18 to 44 years, with mean age of 28 years. Infant’s age ranged from 1 to 7 months, with a mean of 2·5 months. The majority (58 %) of participants were employed full-time and worked in jobs ranging from hairdresser, teacher, trader, seamstress and food vendor (Table 1). Among all participants, the mean household size was six individuals, with most urban and rural participants reporting five members in their household.

**Table 1:** Participant demographics (*n* 38)

Variables	All *n*	%	Rural *n*	%	Urban *n*	%	*P* value
Formal education (years)							
None	3	7·9	2	11·1	1	5·0	0·368
6–9	18	47·4	9	50·0	9	45·0	
10–12	13	34·2	4	22·2	9	45·0	
>12	4	10·5	3	16·7	1	5·0	
Ethnicity							0·001
Akan	17	44·7	12	66·7	5	25·0	
Ga	13	34·2	1	5·6	12	60·0	
Ewe	4	10·5	4	22·2	0	0·0	
Other	4	10·5	1	5·6	3	15·0	
Marital status							0·003
Married	14	36·8	11	61·1	3	15·0	
Cohabitating	24	63·2	7	38·9	17	85·0	
Mother’s employment							0·619
Employed full time	22	57·9	9	50·0	13	65·0	
Employed part time	4	10·5	2	11·1	2	10·0	
Unemployed	12	31·6	7	38·9	5	25·0	
Father’s employment							0·030
Employed full time	32	84·2	13	72·2	19	95·0	
Employed part time	5	13·2	5	27·8	0	0·0	
Unemployed	1	2·6	0	0·0	1	5·0	
Parity							0·046
Primiparous	17	44·7	5	27·8	12	60·0	
Multiparous	21	55·3	13	72·2	8	40·0	
Yearly household income*							0·752
₵<5000	6	15·8	4	22·2	2	10·0	
₵5000–10 000	17	44·7	8	44·4	9	45·0	
₵10 000–₵15 000	7	18·4	3	16·7	4	20·0	
₵ 15 000–20 000	7	18·4	3	16·7	4	20·0	
₵ >20 000	1	2·6	0	0·0	1	5·0	0·752

* ₵ 1 = 0·17 USD, *P*-value tested by *χ*^2^.

Overall, mothers from both rural and urban communities were highly confident in their ability to breast-feed their infant. On a scale of 1–10 with 10 being most confident, the mean response was 8·00 ± 0·35 for rural participants and 8·55 ± 0·39 for urban participants. Out of the thirty-eight participants, twenty-five mothers reported exclusively breast-feeding (EBF) their infant at the time of data collection, with the thirteen remaining participants practicing mixed feeding, which was confirmed by images captured on AIM-2. Because maternal report of type of infant feeding was the same as observation from the captured images, we present results only with maternal report of EBF and mixed feeding. EBF was more common among mothers residing in urban communities (*n* 14, 70 %) compared with their counterparts in the rural communities (*n* 11, 61 %). Mothers with higher household income and longer duration of formal schooling were more likely to EBF their infant, whereas mothers older than 30 years old were less likely to EBF (*n* 9, 60 %) compared with mothers < 30 years old (*n* 16, 69·6 %). A slightly higher number of rural participants (*n* 7, 39 %) reported/captured mixed feeding compared with urban participants (*n* 6, 30 %). Over two-thirds (71 %) of participants reported initiating breast-feeding within the recommended time frame of 1 h after delivery; 12 (66·7 %) of these were from the rural communities, while 15 (75·0 %) were from the urban communities. Mothers from urban communities intended to breast-feed their infant for approximately 4 months longer than rural participants (Urban Mean ± sem: 9·79 ± 1·95 months, Rural Mean ± sem: 5·73 ± 0·27 months).

### Compliance wearing the automatic ingestion monitor device

User compliance with wearing the AIM-2 was generally high among all participants, suggesting acceptability of the device. Mean compliance among the total participants was 83 %. Compliance was higher among urban (86 %) compared with rural (78 %) participants. Urban participants also showed higher overall active time wearing the AIM-2 compared with rural participants. Total ‘active time’ of participant wearing of the AIM-2 among urban mothers was 201 h and 45 min, whereas total ‘active time’ for rural participants was significantly lower at 124 h and 4 min. Total inactive, or non-wear, time was 67 h, with approximately 35 h from rural and 32 h from urban participants. Images from all twenty urban participants were clearer and detectable for annotation compared with images from rural participants. AIM-2 devices worn by two out of the eighteen rural participants had no images captured for reasons unknown, therefore these participants were excluded from the image annotation.

### Relationship between caregiver-reported infant feeding and automatic ingestion monitor-captured images

In general, mothers reported a higher daily breast-feeding frequency than what was observed in the captured AIM images. Similarly, both EBF and mixed feeding mothers reported a longer duration of breast-feeding sessions than from the AIM-2 image observations (Table 2).

**Table 2:** Maternal reported *v*. researcher observed breast-feeding patterns

	Breast-feeding frequency					Breast-feeding duration per session (in seconds)				
	Reported		Observed		*P*-value	Reported		Observed		*P*-value
	Mean	SD	Mean	SD		Mean	SD	Mean	SD	
Rural	9·28	1·42	7·76	0·60	0·446	1517·4	2·95	617·4	0·83	0·909
Urban	12·75	1·27	8·45	0·64	.075	768	2·53	627	1·04	0·003
EBF	13·08	1·26	8·56	0·52	0·167	1058·4	2·77	643·2	0·84	0·468
MF	7·31	0·84	7·25	0·78	0·004	1225·2	3·45	580·2	1·10	0·555

EBF, exclusively breast-feeding; MF, mixed-feeding.

*P*-value tested by *χ*^2^.

Maternal-reported breast-feeding frequency during wake time ranged from 3 to 30 times daily, whereas reported duration of breast-feeding ranged from 2 to 60 min/session. On average, mothers reported mean daily breast-feeding frequency of eleven times, compared with the mean AIM-observed frequency of eight times. Breast-feeding duration was similarly over reported by mothers, with a mean of 18·5 min and AIM-captured mean duration of 10 min/session (Table 2). Accuracy of reporting breast-feeding frequency compared with observations from the images was significantly (*P* = 0·041) higher in the rural community (55·6 %) compared with the urban community (44·4 %). A higher percentage (66·7 %) of mothers who accurately reported breast-feeding duration were from the urban community compared with 33·3 % from the rural community (*P* = 0·007) (Table 2).

### Caregiver-reported breast-feeding experiences and automatic ingestion monitor-captured images

Caregiver-reported breast-feeding experience was ascertained by asking mothers to share their thoughts (in free response) on breast-feeding and through yes/no questions. Urban mothers expressed negative breast-feeding reflections more frequently, whereas all reflections from rural participants were either positive or neutral. Caregiver-reported breast-feeding experiences were categorised into the following common themes: ‘neutral, no issues’ (*n* 12, 33 %), ‘bond or feelings of happiness’ (*n* 14, 39 %), perceived health or financial benefits (*n* 5, 14 %) and ‘complained/expressed struggles’ (*n* 5, 14 %).

Mothers practicing MF were far less confident in the sufficiency of their milk production. Only one mother practicing MF answered ‘yes’ to sufficient milk production, compared with twenty-two mothers practicing EBF. Fifteen out of the thirty-eight (40 %) participants reported that their milk production seemed insufficient attributed to a number of reasons: infant crying (*n* 5, 33 %), infant sleep patterns (*n* 2, 13 %), physical difficulties (*n* 3, 23 %), supplemental feeding (*n* 4, 27 %) and other (*n* 1, 7 %). Infant crying was the most reported reason for perceived insufficient milk.

**Fig. 4:**
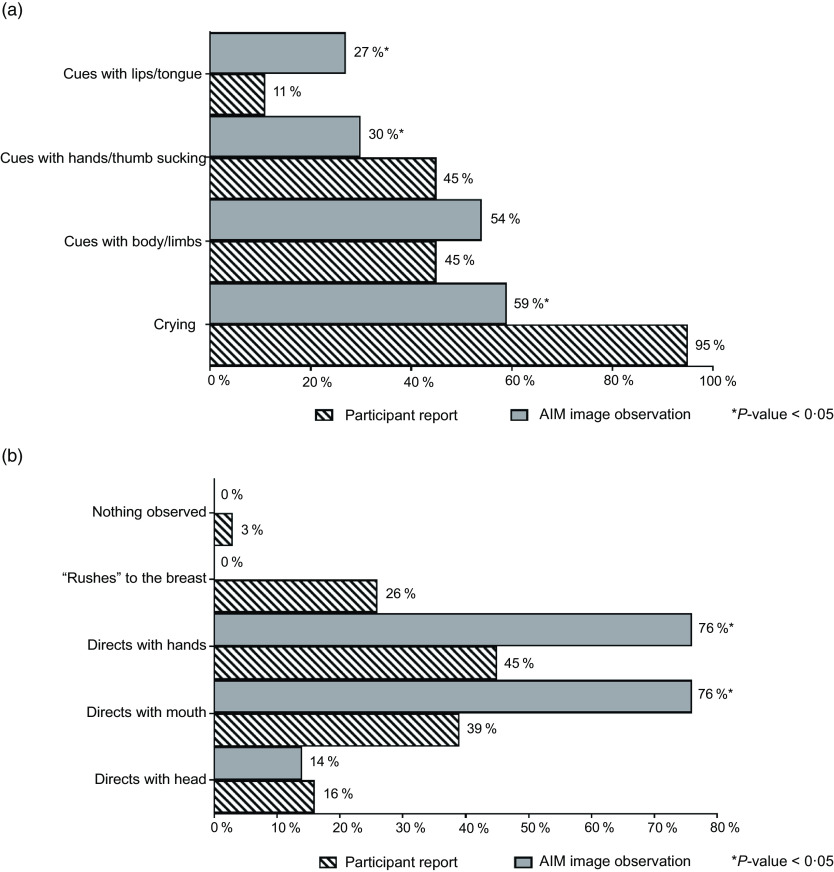
Maternal-reported *v*. researcher-observed infant feeding cues: before breast-feeding session

Feeding cues occurring throughout the middle to end of feeding sessions are reported collectively as ‘during BF session’ (Fig. 3). There was general discrepancy in maternal reported cues for infant expression of enjoyment/satisfaction during feeding compared with observed from captured images (Fig. 3(a)). For expression of infant fullness/satiation during feeding, cues such as nipple removal or refusal to latch and sleep were significantly (*P* < 0·05) underreported by the mother compared with observation from the images (Fig. 3(c)). There was significant (*P* < 0·05) difference between maternal report and observation from the AIM-2 images for sleep and hand/finger movement as cues for satisfaction. Mothers reported diverse range of feeding cues, involving the infants’ head, hands, sounds and facial expressions. Maternal-reported cues for infant expression of hunger included cues with lips/tongue movements, hands/thumb sucking and crying, with significantly (*P* < 0·05) greater disagreement between maternal report and observation from the AIM images (Fig. 4). Maternal report of infant dissatisfaction/disinterest during feeding (Fig. 3(b)) showed the least discrepancy with observations from the captured images, with the exception of head movement.

## Discussion

This is the first study to use the AIM-2 passive dietary assessment device to assess infant feeding practices among a sample of rural and urban mothers in Ghana. Wearable cameras have been used previously among adults and children for dietary assessment^(22,25)^. Studies that have used wearable cameras focused on food and energy intake or portion sizes. In contrast, our study focused on assessing infant feeding habits of mothers with young infants^(32)^. Participants in our study were highly compliant with wearing the AIM-2 during waking hours among both urban and rural communities (86 % and 78 % of waking hours, respectively), making possible the infant feeding assessment from images. As expected, we observed a high degree of discrepancies between maternal report and observations from images captured by the AIM-2, although there was perfect agreement between maternal report and image observation for type of infant feeding.

The current study compared maternal report of infant feeding practices together with digital images captured by the AIM-2 to examine infant feeding habits in rural and urban settings in Ghana. This approach has been suggested as more insightful than each of these methods alone^(32)^. By combining two data collection methods, potential gaps or inaccuracies in memory-based report can be revealed in the digital images, allowing for a higher degree of accuracy and reduction of errors. Notably, accuracy for maternal reported breast-feeding duration was < 30 % for all participants, regardless of their geographic location. Overreporting of breast-feeding duration was higher in the rural communities compared with the urban community, while all underreported duration was detected in the urban communities. One proposed explanation for mothers’ over reported duration is preoccupation with other activities while breast-feeding. The inaccuracies in maternal reports compared with what was passively captured in the images highlight the need for a superior means of infant feeding assessment to accurately examine the nutrition and health of infants to aid appropriate intervention by care providers.

Despite a number of feasibility issues that have been reported in previous wearable device studies^(25)^, our participants showed high compliance with the AIM-2. Total user compliance, or active time wearing the device during wake time, was relatively high, suggesting our participants felt minimal burden wearing the device. Participants were instructed to remove the device during any activities where they preferred privacy; therefore, some non-wear time was expected. The challenging task of manually annotating images by researcher observation as previously noted was similarly experienced in our study^(25)^. The ability to automatically annotate images will go a long way to make wearable devices attractive for dietary and infant feeding assessment at the population level and also in clinical practice.

Although wearable cameras have not been used in infant feeding assessment until now, infant mealtimes have been assessed using third-party video/film^(33–35)^. One major difference between film and digital images is the detection of sounds, such as sucking, which may indicate enjoyment or hunger. We were not able to detect sounds or capture the mother’s behavior while feeding the infant in the current study. A researcher is often present and actively observes the feeding session in film studies; however, in the current study, participants wore the AIM-2 while it passively took pictures during wear-time. The passive nature of capturing the event every 15 s may have reduced bias by either the participant or researcher. Notably, film studies typically only capture 1–2 feeding sessions to lessen the burden on participants^(33,34)^, while our study collected robust wake time data that included at least 9–11 total feeding sessions. Thus, a passive device worn for 1 d allows for a significantly more representative and objective assessment of usual infant feeding patterns compared with the use of active assessment/observation methods such as video or film.

### Strengths and limitations

The current study has several strengths. Most notable was the mixed-methods approach of data collection (self-report and image capture) with a diverse data set. The comparison between two forms of data allowed for a more holistic and objective assessment of each participants’ breast-feeding practices. Inclusion of mothers from both urban and rural Ghanaian communities diversified the data set and allowed us to examine differences based on geographic location. It is possible, however, that questions were misinterpreted in the interview-administered questionnaire. For example, the AIM-2 captured images of breast-feeding sessions during wake-time only, whereas the questionnaire asked mothers for the frequency of breast-feeding ‘each day,’ without ascertaining possible night feedings. This verbiage may have been misinterpreted by mothers as a 24-h period, rather than wake-time only. Because this is a feasibility study using a novel device, some limitations involving the AIM-2's functions were to be expected. The AIM-2 captured images in 15-s intervals, leaving brief gaps in the data collection process. However, the timing of image capture could be altered by reprogramming the firmware, if necessary. The AIM-2 provides digital visual data; thus, cues could be misinterpreted without sounds and live observation. Although the AIM-2 proved to be feasible for infant feeding assessment, it lacks the ability to directly measure the intake of breastmilk. For this reason, particular maternal-reported struggles such as infant dissatisfaction with breastmilk intake and insufficient milk production could not be confirmed using the AIM-2. Lastly, since this was a cross-sectional design and pilot study, the findings should be interpreted with some caution.

## Conclusion

Novel feeding assessment devices such as AIM-2 are feasible and allow for a more individualised, comprehensive and objective assessment of breast-feeding experiences. The AIM-2 data surpass maternal report in accuracy and level of detail. Healthcare professionals who work with new mothers could potentially use this insightful device and data generated to their advantage by further personalising their lactation counselling and support to fit the needs of patients. In addition to healthcare professionals, this device has the potential to improve research and overall assessment of infant feeding habits in relation to infant health. The current study shows the AIM-2 as a feasible and objective alternative to the traditional methods for infant feeding assessment. It provides an objective assessment of infant feeding and has the potential to improve research studies such as those exploring the reasons for cessation of breast-feeding. Increasing the rates and duration of breast-feeding is a top priority of public health authorities as there a clear health benefits for mothers and offspring, hence the device can ultimately help improve the infant health. However, future technological advances, which may eliminate the time-consuming, manual image annotation, will extend the applicability of the device on an even larger scale.

## References

[1] Victora CG , Bahl R , Barros AJD et al. (2016) Breastfeeding in the 21st century: epidemiology, mechanisms, and lifelong effect. Lancet 387, 475–490. 26869575 10.1016/S0140-6736(15)01024-7

[2] Feltner C , Weber RP , Stuebe A et al. (2018) Breastfeeding Programs and Policies, Breastfeeding Uptake, and Maternal Health Outcomes in Developed Countries. Rockville, MD: Agency for Healthcare Research and Quality (US). 30204377

[3] Quesada JA , Méndez I & Martín-Gil R (2020) The economic benefits of increasing breastfeeding rates in Spain. Int Breastfeed J 15, 34. 32366305 10.1186/s13006-020-00277-w PMC7197147

[4] Chowdhury R , Sinha B , Sankar MJ et al. (2015) Breastfeeding and maternal health outcomes: a systematic review and meta-analysis. Acta Paediatr 104, 96–113. 26172878 10.1111/apa.13102 PMC4670483

[5] Ip S , Chung M , Raman G et al. (2007) Breastfeeding and maternal and infant health outcomes in developed countries. Evid Rep Technol Assess 153, 1–186. PMC4781366 17764214

[6] Centers for Disease Control and Prevention (2019) Breastfeeding: Why It Matters. https://www.cdc.gov/breastfeeding/about-breastfeeding/why-it-matters.html (accessed March 2021).

[7] Barclay AR , Russell RK , Wilson ML et al. (2009) Systematic review: the role of breastfeeding in the development of pediatric inflammatory bowel disease. J Pediatr 155, 421–426. 19464699 10.1016/j.jpeds.2009.03.017

[8] UNICEF (2018) Breastfeeding, A Mother’s Gift, for Every Child. https://www.unicef.org/publications/files/UNICEF_Breastfeeding_A_Mothers_Gift_for_Every_Child.pdf (accessed February 2021).

[9] Herba CM , Roza S , Govaert P et al. (2013) Breastfeeding and early brain development: the Generation R study. Matern Child Nutr 9, 332–349. 23167730 10.1111/mcn.12015 PMC6860584

[10] Kavle JA , LaCroix E , Dau H et al. (2017) Addressing barriers to exclusive breast- feeding in low- and middle-income countries: a systematic review and programmatic implications. Public Health Nutr 20, 3120–3134. 28965508 10.1017/S1368980017002531 PMC10262277

[11] World Health Organization & United Nations Children’s Fund (2019) Global Breastfeeding Scorecard, 2019: Increasing Commitment to Breastfeeding through Funding and Improved Policies and Programmes. https://apps.who.int/iris/handle/10665/326049 (accessed January 2021).

[12] Kakute PN , Ngum J , Mitchell P et al. (2005) Cultural barriers to exclusive breastfeeding by mothers in a rural area of Cameroon. Afr J Midwifery Womens Health 50, 324–328. 10.1016/j.jmwh.2005.01.005 15973270

[13] Gatti L (2008) Maternal perceptions of insufficient milk supply in breastfeeding. J Nurs Scholarsh 40, 355–363. 19094151 10.1111/j.1547-5069.2008.00234.x PMC4508856

[14] Neifert MR (2001) Prevention of breastfeeding tragedies. Pediatr Clin North Am 48, 273–297. 11339153 10.1016/s0031-3955(08)70026-9

[15] Morrison AH , Gentry R & Anderson J (2019) Mothers’ reasons for early breastfeeding cessation. MCN Am J Matern Child Nurs 44, 325–330. 31633522 10.1097/NMC.0000000000000566

[16] Hornsby PP , Gurka KK , Conaway MR et al. (2019) Reasons for early cessation of breastfeeding among women with low income. Breastfeed Med 14, 375–381. 30994371 10.1089/bfm.2018.0206

[17] Odom EC , Li R , Scanlon KS et al. (2013) Reasons for earlier than desired cessation of breastfeeding. Pediatrics 131, e726–e732. 23420922 10.1542/peds.2012-1295 PMC4861949

[18] Macdiarmid J & Blundell J (1998) Assessing dietary intake: who, what and why of under-reporting. Nutr Res Rev 11, 231–253. 19094249 10.1079/NRR19980017

[19] Schatzkin A , Kipnis V , Carroll RJ et al. (2003) A comparison of a food frequency questionnaire with a 24-h recall for use in an epidemiological cohort study: results from the biomarker-based observing protein and energy nutrition (OPEN) study. Int J Epidemiol 32, 1054–1062. 14681273 10.1093/ije/dyg264

[20] Ravelli MN & Schoeller DA (2020) Traditional self-reported dietary instruments are prone to inaccuracies and new approaches are needed. Front Nutr 7, 90. 32719809 10.3389/fnut.2020.00090 PMC7350526

[21] Fisher JO , Butte NF , Mendoza PM et al. (2008) Overestimation of infant and toddler energy intake by 24-h recall compared with weighed food records. Am J Clin Nutr 88, 407–415. 18689377 10.1093/ajcn/88.2.407

[22] Anderson A , McCrory M , Steiner-Asiedu M et al. (2019) A case study of household food-related assessment using an innovative passive dietary assessment device in Mampong-Akuapem, Ghana (FS17–03–19). Curr Dev Nutr 3, Suppl. 1, nzz035.

[23] Doulah A , Ghosh T , Hossain D et al. (2021) Automatic ingestion monitor version 2 – a novel wearable device for automatic food intake detection and passive capture of food images. IEEE JBHI 25, 568–576. 10.1109/JBHI.2020.2995473 PMC7938421 32750904

[24] Jobarteh ML , McCrory MA , Lo B et al. (2020) Development and validation of an objective, passive dietary assessment method for estimating food and nutrient intake in households in low- and middle-income countries: a study protocol. Curr Dev Nutr 4, nzaa020. doi: 10.1093/cdn/nzaa020. 32099953 PMC7031207

[25] Beltran A , Dadabhoy H , Ryan C et al. (2018) Dietary assessment with a wearable camera among children: feasibility and intercoder reliability. J Acad Nutr Diet 118, 2144–2153. 30115556 10.1016/j.jand.2018.05.013 PMC8114336

[26] Raber M , Patterson M , Jia W et al. (2018) Utility of eButton images for identifying food preparation behaviors and meal-related tasks in adolescents. Nutr J 17, 32. 29477143 10.1186/s12937-018-0341-2 PMC6389239

[27] World Health Organization & UNICEF (2018) Early Initiation of Breastfeeding. https://www.who.int/news/item/11-04-2018-who-and-unicef-issue-new-guidance-to-promote-breastfeeding-in-health-facilities-globally (accessed March 2021).

[28] Li R , Fein SB , Chen J et al. (2008) Why mothers stop breastfeeding: mothers’ self- reported reasons for stopping during the first year. Pediatrics 122, Suppl. 2, S69–S76. 18829834 10.1542/peds.2008-1315i

[29] Feenstra MM , Jørgine Kirkeby M , Thygesen M et al. (2018) Early breastfeeding problems: a mixed method study of mothers’ experiences. Sex Reprod Healthc 16, 167–174. 29804762 10.1016/j.srhc.2018.04.003

[30] Llewellyn CH , van Jaarsveld CHM , Johnson L et al. (2011) Development and factor structure of the baby eating behaviour questionnaire in the Gemini birth cohort. Appetite 57, 388–396. 21672566 10.1016/j.appet.2011.05.324

[31] Cerminaro C , Sazonov E , McCrory MA et al. (2021) The use of passive dietary assessment among breastfeeding mothers in Ghana. Msc Thesis, The University of Georgia.

[32] O’Loughlin G , Cullen SJ , McGoldrick A et al. (2013) Using a wearable camera to increase the accuracy of dietary analysis. Am J Prev Med 44, 297–301. 23415128 10.1016/j.amepre.2012.11.007

[33] Shloim N , Vereijken CMJL , Blundell P et al. (2017) Looking for cues – infant communication of hunger and satiation during milk feeding. Appetite 108, 74–82. 27647500 10.1016/j.appet.2016.09.020

[34] Nekitsing C , Madrelle J , Barends C et al. (2016) Application and validation of the feeding infants: behaviour and facial expression coding system (FIBFECS) to assess liking and wanting in infants at the time of complementary feeding. Food Qual Prefer 48, 228–237.

[35] Hetherington MM (2017) Understanding infant eating behaviour – lessons learned from observation. Physiol Behav 176, 117–124. 28089707 10.1016/j.physbeh.2017.01.022

